# The efficiency of aspheric intraocular lens according to biometric measurements

**DOI:** 10.1371/journal.pone.0182606

**Published:** 2017-10-16

**Authors:** Woong-Joo Whang, Junjie Piao, Young-Sik Yoo, Choun-Ki Joo, Geunyoung Yoon

**Affiliations:** 1 Department of Ophthalmology and Visual Science, Seoul St. Mary's Hospital, College of Medicine, The Catholic University of Korea, Seoul, Korea; 2 Flaum Eye Institute, Center for Visual Science, The Institute of Optics, University of Rochester, Rochester, New York, United States of America; Bascom Palmer Eye Institute, UNITED STATES

## Abstract

**Purpose:**

To analyze internal spherical aberration in pseudophakic eyes that underwent aspheric intraocular lens (IOL) implantation, and to investigate the relationships between biometric data and the effectiveness of aspheric IOL implantation.

**Methods:**

This retrospective study included 40 eyes of 40 patients who underwent implantation of an IOL having a negative spherical aberration of -0.20 μm (CT ASPHINA 509M; Carl Zeiss Meditec Inc., Germany). The IOLMaster (version 5.0; Carl Zeiss AG, Germany) was used for preoperative biometric measurements (axial length, anterior chamber depth, central corneal power) and the measurement of postoperative anterior chamber depth. The spherical aberrations were measured preoperatively and 3 months postoperatively using the iTrace (Tracey Technologies, Houston, TX, USA) at a pupil diameter of 5.0 mm. We investigated the relationships between preoperative biometric data and postoperative internal spherical aberration, and compared biometric measurements between 2 subgroups stratified according to internal spherical aberration (spherical aberration ≤ -0.06 μm vs. spherical aberration > -0.06 μm).

**Results:**

The mean postoperative internal spherical aberration was -0.087 ± 0.063 μm. Preoperative axial length and residual total spherical aberration showed statistically significant correlations with internal spherical aberration (*p* = 0.041, 0.002). Preoperative axial length, postoperative anterior chamber depth, IOL power, and residual spherical aberration showed significant differences between the 2 subgroups stratified according to internal spherical aberration (*p* = 0.020, 0.029, 0.048, 0.041 respectively).

**Conclusion:**

The corrective effect of an aspheric IOL is influenced by preoperative axial length and postoperative anterior chamber depth. Not only the amount of negative spherical aberration on the IOL surface but also the preoperative axial length should be considered to optimize spherical aberration after aspheric IOL implantation.

## Introduction

The goal of modern cataract surgery is not only to improve visual acuity but also to provide the best quality of vision possible. The virgin cornea has a positive spherical aberration, [1] and corneal aberrations are at least partially compensated for by lenticular aberrations in young human eyes. However, positive spherical aberrations of the cornea increase with age while the lens loses the ability to compensate for the aberrations. [2] The aspheric intraocular lens (IOL) was developed to compensate for corneal aberrations and previous studies demonstrated the functional vision benefits of aspheric IOLs over conventional spherical IOLs. [3–5]

A pseudophakic eye acts as a 2-lens system consisting of the cornea and the IOL, which project images onto the retina. Corneal aberration measured by a ray-tracing type aberrometer is developed by the anterior corneal surface, while internal spherical aberration is defined as the aberration induced by 2 elements: the posterior corneal surface and an IOL. As the spherical aberration from the posterior corneal surface is subtle, internal spherical aberration is mainly affected by an IOL.

The distance between the 2 lenses (the cornea and IOL) is called the effective lens position (ELP) or postoperative anterior chamber depth. The ELP affects both the IOL spherical power for correction of the spherical equivalent and the cylinder power for reducing the postoperative refractive cylinder in toric IOL implantation. [6] A longer axial length and deeper ELP also reduced the effect of near add power of a multifocal IOL. [7] Based on the results of recent studies, we hypothesized that the effectiveness of spherical aberration correction in the corneal plane would vary according to preoperative biometry and postoperative anterior chamber depth in patients undergoing aspheric IOL insertion.

Therefore, the purpose of this study was to investigate the relationships between biometric data (preoperative axial length, anterior chamber depth, corneal power, postoperative anterior chamber depth, and IOL power) and the internal spherical aberration after aspheric IOL implantation, and to analyze the effect of an aspheric IOL according to biometric data.

## Methods

This retrospective study included 40 eyes of 40 patients who had undergone cataract surgery between March 2015 and May 2015. Informed consent was verbal and obtained from all the patients prior to the commencement of the study, and the study methods adhered to the tenets of the Declaration of Helsinki for use of human participants in biomedical research. The Institutional Review Board for Human Studies at Seoul St. Mary’s Hospital approved this study and waived the written form of informed consent for this retrospective study. None of the patients had a history of ocular disease, previous ocular surgery, or general disorders affecting the cornea and there were no intraoperative and postoperative complications.

The IOLMaster (version 5.0; Carl Zeiss Meditec, Jena, Germany) was uses partial coherence interferometry to measure axial length, was used for preoperative biometric measurements. Corneal power was measured by automated keratometry, which was performed first because the system requires the input of corneal radii to calculate the anterior chamber depth. The anterior chamber depth was determined preoperatively and 3 months postoperatively by calculating the distance along the visual axis between the corneal epithelium and the anterior lens surface using lateral slit illumination.

After preoperative measurements were made, all patients underwent cataract surgery via a 2.2-mm micro-coaxial incision. All surgeries were performed using the OZil torsional handpiece with the INFINITI Vision System (Alcon, USA). All procedures were performed by the same surgeon (C. K. Joo). Local anaesthesia was administered using topical 4% lidocaine and 0.5% proparacaine hydrochloride (Alcaine, Alcon, USA). Surgery was performed through a self-sealing, temporal clear corneal incision. The Intrepid ClearCut 2.2-mm dual-bevel metal keratome (Alcon) was used to make the incisions. The Akahoshi Phaco PreChopper (Katena Eye Inc., USA) was used to fracture the nucleus in half. The cataract was removed through the 2.2-mm micro-coaxial incision using a 0.9 mm mini flared 30° reverse-Kelman ABS tip. Phacoemulsification was performed with a 100% torsional ultrasound, a 350 mm Hg vacuum, and an aspiration rate of 35 mL/min.

Following phacoemulsification, the same type of IOL (CT ASPHINA 509M; Carl Zeiss Meditec Inc., Germany) was inserted into the capsular bag in each patient. No intraoperative complications occurred. The CT ASPHINA 509M has an aspheric design to counterbalance corneal aspherical aberration, with a negative spherical aberration of -0.20 μm. We applied optimized IOL constants published on the User Group for Laser Interference Biometry website (http://www.augenklinik.uni-wuerzburg.de/ulib/c1.htm).

The spherical aberrations were measured preoperatively and 3 months postoperatively using the iTrace (Tracey Technologies, Houston, TX, USA) at a pupil size of 5.0 mm, after pharmacological dilatation with Mydrin-P (Santen Pharmaceutical, Osaka, Japan). The residual total spherical aberration was defined as preoperative spherical aberration minus postoperative spherical aberration. The iTrace is a ray tracing-type aberrometer and assesses aberrations using a detector to measure the displacement of a laser beam reflected from the retina.

**Fig 1:**
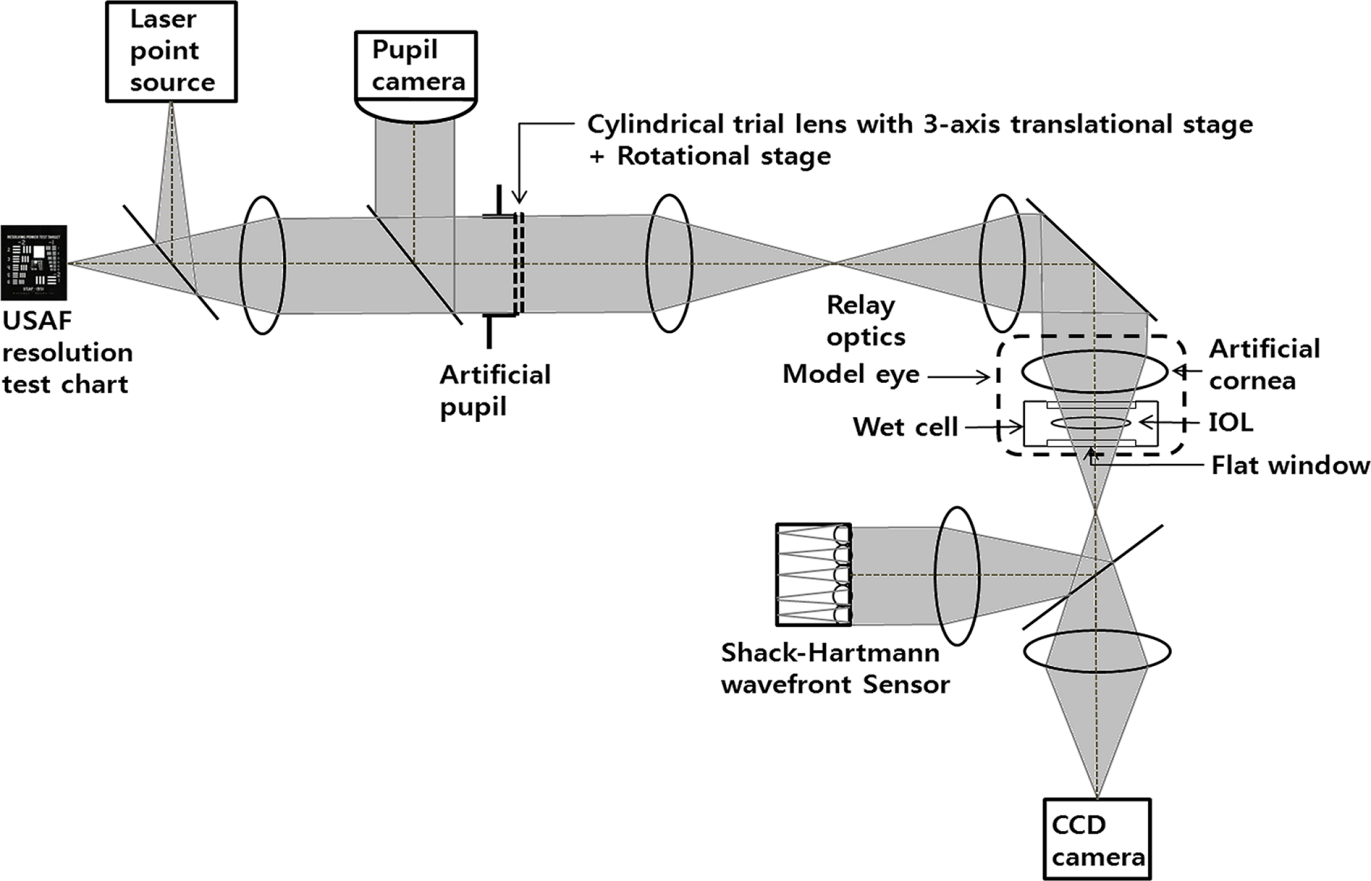
Diagram of the optical bench metrology system used.

To investigate whether the negative spherical aberration of an IOL differs according to IOL diopter, we performed additional optical bench testing and analyzed the internal spherical aberrations of 4 different IOL diopters. CT ASPHINA 509M IOLs with diopters of 1, 11, 21, and 31 were used in the optical bench testing. Fig 1 shows a schematic diagram of the optical bench metrology system used in this study. The system consisted of a model eye, an artificial pupil, a pupil camera, the 1951 United States Air Force (1951 USAF) resolution test chart, a charge-coupled device (CCD) camera (Guppy pro F503C; ALLIED Vision Technologies GmbH, Stadtroda, Germany), and a Hartmann-Shack wavefront sensor (WFS150-5C; Thorlabs Inc., Newton, NJ, USA). The model eye comprised a wet cell mounting on the XYZ translation stage for precise alignment. The front and back surfaces of the wet cell were clear flat windows. The IOL was positioned inside the wet cell and was filled with balanced salt solution, which had a refractive index similar to that of aqueous humour. The artificial pupil was fixed at a size of 5 mm and positioned in the pupil plane. It could be seen from above the IOL, through the relay optics. Since the pupil camera showed images of the artificial cornea, artificial pupil, and IOL simultaneously, the precise pupil diameter was determined in real-time with the pupil camera and IOL alignment was possible. The 1951 USAF resolution chart was used as the resolution target and positioned in the retina image plane. The chart was illuminated by white light, and a CCD camera acquired images. In addition, changes in wavefront aberrations (low and high order aberrations) of aspheric IOLs were measured with the Hartmann-Shack wavefront sensor using a 635 nm-wavelength laser diode (OZ Optics Ltd., Ottawa, Canada).

Linear regression analysis and Pearson correlation tests were performed to determine the relationships between biometric data (preoperative axial length, anterior chamber depth, central corneal power, postoperative anterior chamber depth, IOL power, preoperative total spherical aberration, postoperative total spherical aberration, and residual total spherical aberration) and the internal spherical aberration measurements obtained using the ray tracing aberrometer. The biometric measurements and implanted IOL power were compared between 2 subgroups stratified according to internal aberration (spherical aberration ≤ -0.06 μm vs. spherical aberration > -0.06 μm). To determine the significance of the differences in measurements between the 2 subgroups, a Mann-Whitney U test was performed. Statistical analyses were performed using Statistical Package for the Social Sciences (SPSS) software (version 21.0; SPSS, Inc., USA) and the total experimental level of significance was set at 0.05.

## Results

**Fig 2:**
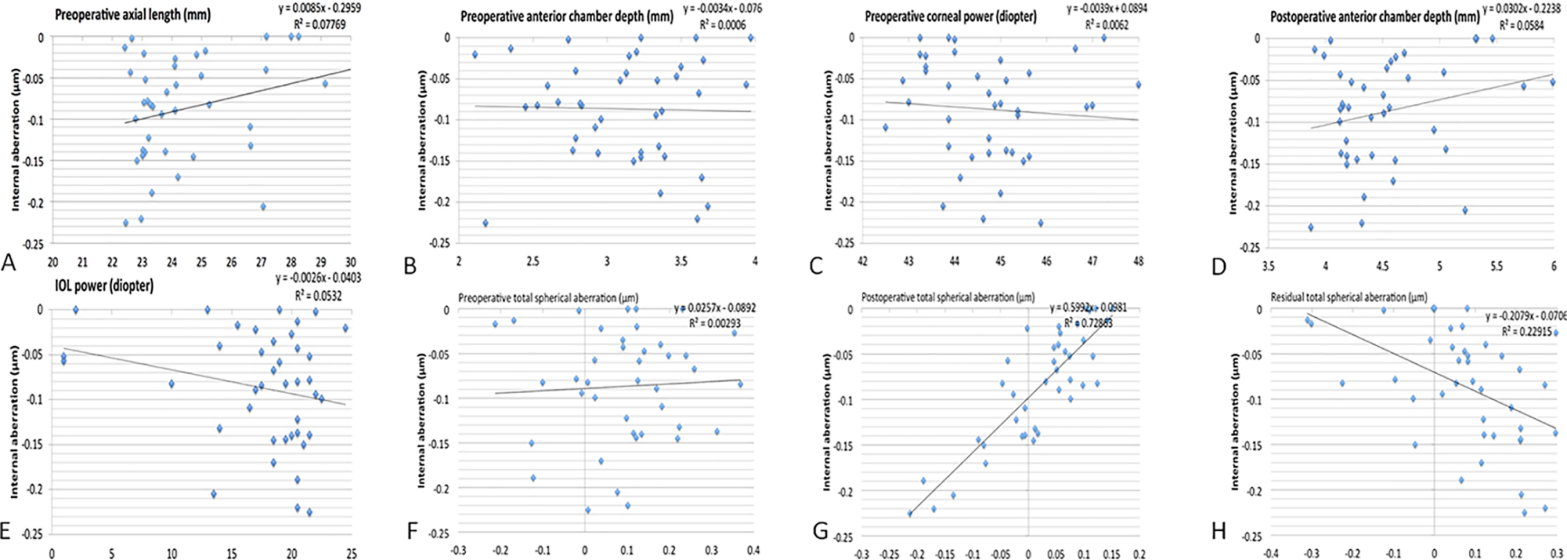
Linear regression analysis of the relationships between biometry parameter and spherical aberration. (A) Preoperative axial length and internal spherical aberration. (B) Preoperative anterior chamber depth and internal spherical aberration. (C) Preoperative central corneal power and internal spherical aberration. (D) Postoperative anterior chamber depth (effective lens position) and internal spherical aberration. (E) Implanted IOL power and internal spherical aberration. (F) Preoperative total spherical aberration and internal spherical aberration. (G) Postoperative total spherical aberration and internal spherical aberration. (H) Residual total spherical aberration and internal spherical aberration.

The IOLMaster biometry and postoperative ray tracing aberrometer data were available for all 40 eyes of the 40 patients included in the study. Seventy-five percent (30 eyes) of the study population consisted of women. S1 Table shows patient age at surgery, preoperative biometric measurements (axial length, anterior chamber depth, central corneal power, total spherical aberration), postoperative anterior chamber depth, IOL power, and postoperative total and internal spherical aberration. Mean postoperative internal spherical aberration was -0.087 ± 0.063 μm. Fig 2 demonstrates the relationships between the biometric measurements and internal spherical aberration. Preoperative axial length showed a statistically significant correlation with internal spherical aberration (*r* = 0.279, *p* = 0.041). Both the postoperative total spherical aberration and the residual total spherical aberration were also significantly correlated with internal spherical aberration (*r* = 0.854, *p* < 0.001; *r* = -0.479, *p* = 0.002).

On the other hand, preoperative anterior chamber depth, preoperative central corneal power, preoperative total spherical aberration, postoperative anterior chamber depth and IOL power were not significantly correlated with internal spherical aberration (*r* = -0.025, *p* = 0.440; *r* = -0.079, *p* = 0.315; *r* = 0.280, *p* = 0.081; *r* = 0.242, *p* = 0.133; *r* = -0.231, *p* = 0.076, respectively).

The analysis of subgroups stratified according to internal spherical aberration is shown in S2 Table. Preoperative axial length, postoperative anterior chamber depth and IOL power showed significant differences between the 2 groups. The low efficiency group (internal spherical aberration > -0.06 μm) included eyes with longer axial length, deeper IOL position, lower IOL power, higher postoperative total spherical aberration, and lower residual total spherical aberration (*p* = 0.017, 0.048, <0.001, 0.041, respectively).

**Fig 3:**
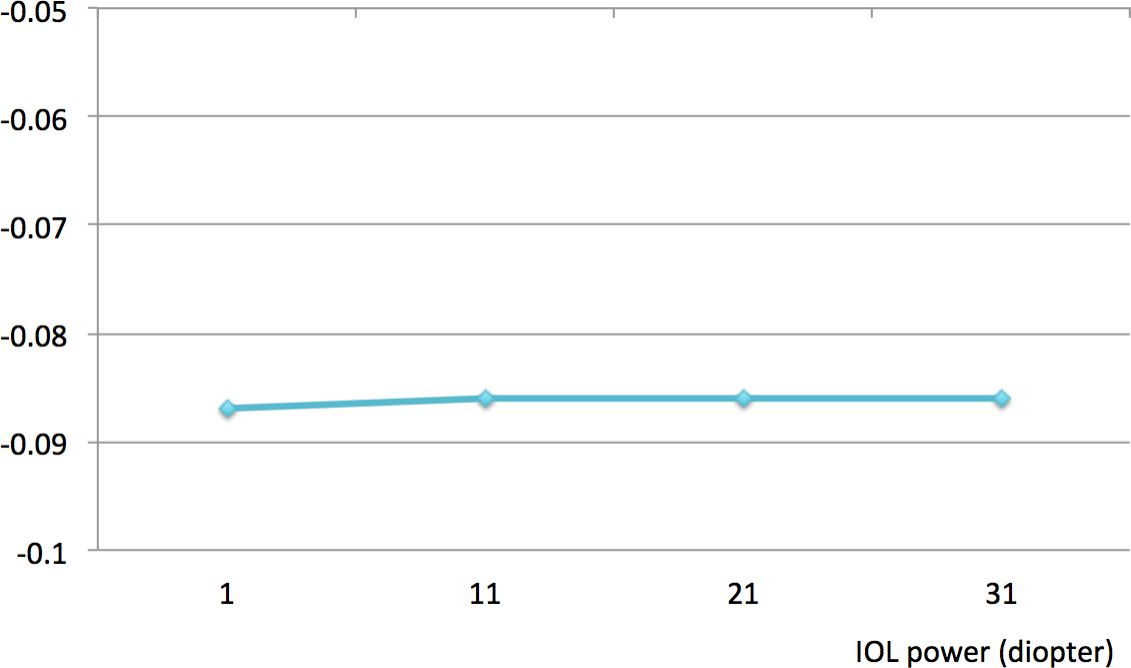
Spherical aberrations derived from optical bench testing.

Fig 3 demonstrates the spherical aberrations according to IOL diopter. Spherical aberrations in IOLs of 1, 11, 21, and 31 diopters in a 5 mm pupil were -0.087 μm, -0.086 μm, -0.086 μm, and -0.086 μm, respectively. Regardless of IOL diopter, spherical aberrations were constant in optical bench testing and the values were the same as those provided by the manufacturer. Based on the results of optical bench testing, we concluded that changes in IOL diopter are not very likely to affect internal spherical aberration.

## Discussion

The difference in spherical aberration is the main factor that has been used to demonstrate the relative superiority of aspheric IOLs. Previous studies have not investigated the relationship between biometric measurements and the efficiency of aspheric IOL implantation. This is the first study investigating factors affecting internal spherical aberration in eyes with an aspheric IOL. Based on the results of this study, we can assume that IOL power, preoperative axial length, and postoperative IOL position are contributing factors that determine the efficiency of aspheric IOL in spherical aberration correction.

Although IOL diopter was not significantly correlated with internal spherical aberration, there was a statistically significant difference in IOL diopter in the subgroup analysis according to internal spherical aberration.

However, spherical aberrations were constant in optical bench testing and, we concluded that changes in IOL diopter are less likely to affect internal spherical aberration.

Preoperative axial length and postoperative anterior chamber depth were other potential factors influencing internal spherical aberration. In subgroup analyses stratified according to internal spherical aberration, there were significant differences in preoperative axial length and postoperative anterior chamber depth between the groups. Axial length was positively correlated with internal spherical aberration. A study, by Savini et al. [8] demonstrated that the postoperative anterior chamber depth influenced the ratio between the cylinder power in the IOL plane and the cylinder power in the corneal plane. Overcorrection may occur in eyes with a flat corneal power and short axial length, while under correction can occur in eyes with a steep corneal power and long axial length. Therefore, the correction ratio of toric IOLs is smaller in longer eyes with the same corneal power. They also calculated the effective adding power of a multifocal IOL and concluded that the adding power on corneal plane also changed according to preoperative corneal power and axial length. [7] Shorter eyes with flatter corneas showed relatively higher adding powers on corneal plane and longer eyes with steeper corneas had lower adding powers. Since there was no difference in preoperative corneal power in the subgroup analysis of our study, a relatively higher efficiency of aspheric IOL implantation may occur in shorter eyes while a lower efficiency may occur in longer eyes on the same principle. These results are caused by the relationship between IOL position and axial length.

A spherical aberration occurs when the refraction of light rays in the central areas of the cornea differs from the refraction of light rays in the periphery. In normal corneas, the anterior surface has positive spherical aberration and the focal point of peripheral rays lies in front of the focal point of central rays. [9] The aspheric IOL was designed with an aspheric surface that induced negative spherical aberrations and offset positive spherical aberration derived from the anterior cornea. The reduction of spherical aberration after aspheric IOL implantation has been shown to improve vision and contrast sensitivity compared with those of the spherical IOL implantation. [10,11] In the current study, we applied the CT ASPHINA 509M IOL, which has a negative spherical aberration of -0.18 μm in.

In previous studies assessing the differences between aspheric IOLs and spherical IOLs, statistically significant differences in spherical aberrations were found for all pupil diameters. [12] As pupil diameter increased, the absolute value of internal spherical aberration in eyes with an aspheric IOL increased. [13] This also indicates that the efficiency of aspheric IOLs changes according to the change in pupil diameter. A study by Eom et al. [14] found a negative correlation between internal spherical aberration and pupil diameter after aspheric IOL implantation. They calculated mathematically that minimum pupil diameters of 3.4–3.7 mm were effective to compensate for positive spherical aberration of the cornea. In the present study, we only evaluated a fixed pupil diameter of 5.0 mm.

In the current study, we used a ray tracing-type aberrometer. This equipment uses sequential beams of light passing into the eye and senses the exact positions where these beams reach. Since we measured ocular aberrations in a 5.0 mm zone, approximately 178 points were included for spherical aberration analysis in the present study. Ray tracing-type aberrometers have demonstrated repeatability when measuring high-order aberrations, including spherical aberration. [15,16] In addition, the ray tracing method shows a higher success rate for aberration measurements. In particular, it is more suitable for measurements in highly aberrant eyes than the Hartmann-Shack method, which uses a lenslet array to obtain a large number of points. [17]

The current study has some limitations. First, internal spherical aberration measurements using the ray tracing aberrometer included spherical aberration on the posterior corneal surface. Since the refractive index difference between the posterior cornea and aqueous humour is approximately 10.6% of the difference between the air and anterior cornea, the spherical aberration on the posterior corneal surface is expected to be subtle. Corneal aberration measurements using the Scheimpflug rotating camera, which measures high-order aberrations on the posterior corneal surface, will helpful enhance the accuracy of internal spherical aberration analysis in the future. Second, we did not consider the optical effect resulting from IOL tilt or decentration. In the previous studies, the critical amount of IOL decentration and tilt were 0.4–0.8 mm and tilt 5–10 degrees, respectively. [18,19] An accurate evaluation of IOL decentration or tilt and exclusion of cases with excessive decentration or tilt will be necessary in the future studies.

In conclusion, internal spherical aberration after aspheric IOL implantation is influenced by preoperative axial length. Cataract surgeons should recognize that the efficiency of an aspheric IOL will be reduced after cataract surgery in longer eyes and that they may be able to predict postoperative state from preoperative axial length.

## Supporting information

S1 Table Demographic data. (DOCX)

S2 Table Preoperative biometric data (axial length, anterior chamber depth, and central corneal power) and implanted IOL power in 2 subgroups stratified by internal spherical aberration. (DOCX)

## References

[1] Oshika T, Klyce SD, Applegate RA, Howland HC (1999) Changes in corneal wavefront aberrations with aging. Invest Ophthalmol Vis Sci 40:1351–1355. 10359316

[2] Hofer H, Artal P, Singer B, Aragon JL, Williams DR (2001) Dynamics of the eye’s wave aberration. J Opt Soc Am A Opt Image Sci Vis 18:497–506. 11265680 10.1364/josaa.18.000497

[3] Denoyer A, Le Lez ML, Majzoub S, Pisella PJ (2007) Quality of vision after cataract surgery after Tecnis Z9000 intraocular lens implantation: effect of contrast sensitivity and wavefront aberration improvements on the quality of daily vision. J Cataract Refract Surg 33:210–216. doi: 10.1016/j.jcrs.2006.10.035 17276260 10.1016/j.jcrs.2006.10.035

[4] Bellucci R, Scialdone A, Buratto L, Morselli S, Chierego C, Criscuoli A, et al (2005) Visual acuity and contrast sensitivity comparison between Tecnis and AcrySof SA60AT intraocular lenses: A multicenter randomized study. J Cataract Refract Surg 31:712–717. doi: 10.1016/j.jcrs.2004.08.049 15899447 10.1016/j.jcrs.2004.08.049

[5] Bellucci R, Morselli S, Pucci V (2007) Spherical aberration and coma with an aspherical and a spherical intraocular lens in normal age-matched eyes. J Cataract Refract Surg 33:203–209. doi: 10.1016/j.jcrs.2006.10.068 17276259 10.1016/j.jcrs.2006.10.068

[6] Eom Y, Kang SY, Song JS, Kim YY, Kim HM (2015) Effect of effective lens position on cylinder power of toric intraocular lenses. Can J Ophthalmol 50:26–32. doi: 10.1016/j.jcjo.2014.08.003 25677279 10.1016/j.jcjo.2014.08.003

[7] Savini G, Hoffer KJ, Lombardo M, Serrao S, Schiano-Lomoriello D, Ducoli P (2016) Influence of the effective lens position, as predicted by axial length and keratometry, on the near add power of mulifocal intraocular lenses. J Cataract Refract Surg 41:44–49. doi: 10.1016/j.jcrs.2015.07.044 PMID: 26948777 10.1016/j.jcrs.2015.07.044 26948777

[8] Savini G, Hoffer KJ, Carbonelli M, Ducoli P, Barboni P (2013) Influence of axial length and corneal power on the astigmatisc power of toric intraocular lenses. J Cataract Refract Surg 39:1900–1903. 24427798 10.1016/j.jcrs.2013.04.047

[9] Schuster AK, Tesarz J, Vossmerbaeumer U (2015) Ocular wavefront analysis of aspheric compared with spherical monofocal intraocular lenses in cataract surgery: Sysematic review with metaanalysis. J Cataract Refract Surg 41:1088–1097. doi: 10.1016/j.jcrs.2015.04.005 25956711 10.1016/j.jcrs.2015.04.005

[10] Schuster AK, Tesarz J, Vosserbaeumer U (2013) The impact on vision of aspheric to spherical monofocal intraocular lenses in cataract surgery: a systematic review with meta-analysis. Ophthalmology 120:2166–2175. doi: 10.1016/j.ophtha.2013.04.011 23751220 10.1016/j.ophtha.2013.04.011

[11] Kasper T, Buhren J, Kohnen T (2006) Visual performance of aspherical and spherical intraocular lenses: intraindividual comparison of visual acuity, contrast sensitivity, and higher-order aberrations. J Cataract Refract Surg 32:2022–2029. doi: 10.1016/j.jcrs.2006.07.029 17137978 10.1016/j.jcrs.2006.07.029

[12] Crnej A, Buehl W, Greslechner R, Hirnschall N, Findl O. Effect of an aspheric intraocular lens on the ocular wave-front adjusted for pupil size and capsulorhexis size. Acta Ophthalmol 92:e353–357. doi: 10.1111/aos.12344 24479668 10.1111/aos.12344

[13] Jun I, Choi YJ, Kim EK, Seo KY, Kim TI (2012) Internal spherical aberration by ray tracing-type aberrometry in multifocal pseudophakic eyes. Eye 26:1243–1248. doi: 10.1038/eye.2012.129 22744386 10.1038/eye.2012.129 PMC3443835

[14] Eom Y, Yoo E, Kang SY, Kim HM, Song JS (2013) Change in efficiency of aspheric intraocular lenses based on pupil diameter. Am J Ophthalmol 155:492–498.e2 doi: 10.1016/j.ajo.2012.09.024 23218695 10.1016/j.ajo.2012.09.024

[15] Visser N, Berendschot TT, Verbakel F, Tan AN, de Brabander J, Nuijts RM (2011) Evaluation of the comparability and repeatability of four wavefront aberrometers. Invest Ophthalmol Vis Sci 52:1302–1311. doi: 10.1167/iovs.10-5841 21051697 10.1167/iovs.10-5841

[16] Bartsch DU, Bessho K, Gomez L, Freeman WR (2008) Comparison of laser ray-tracing and skiascopic ocular wavefront-sensing devices. Eye 22:1384–1390. doi: 10.1038/sj.eye.6702901 17571088 10.1038/sj.eye.6702901 PMC2666015

[17] Wang L, Wang N, Koch DD (2003) Evaluation of refractive error measurements of the Wavescan Wavefront system and the Tracey Wavefront aberrometer. J Cataract Refract Surg 29:970–979. 12781285 10.1016/s0886-3350(02)01967-3

[18] Holladay JT, Piers PA, Koranyi G, van der Mooren M, Norrby NE (2002) A new intraocular lens design to reduce spherical aberration of pseudophakic eyes. J Refract Surg 18:683–691. 12458861 10.3928/1081-597X-20021101-04

[19] Piers PA, Weeber HA, Artal P, Norrby S (2007) Theoretical comparison of aberration-correction customized and aspheric intraocular lenses. J Refract Surg 23:374–384. . 17455833 10.3928/1081-597X-20070401-10

