# Supplementary material for: The efficiency of aspheric intraocular lens according to biometric measurements
Source: PLoS One. 2017 Oct 16;12(10):e0182606. doi: 10.1371/journal.pone.0182606 (PMC5642893; doi:10.1371/journal.pone.0182606)
Supplement: S1 Table — (DOCX) [file pone.0182606.s001.docx]

| Variable | Value |
| --- | --- |
| Age (years)  Mean $\pm$ SD  Range | 63.30 ± 10.64  (40—83) |
| Laterality  OD  OS | 21 (52.5%)  19 (47.5%) |
| Preoperative axial length (mm) | 24.53 ± 2.07 (22.42 ~ 30.97) |
| Preoperative anterior chamber depth (mm) | 3.18 ± 0.46 (2.11 ~ 3.97) |
| Preoperative central corneal power (diopters) | 44.72 ± 1.26 (42.50 ~ 48.00) |
| Preoperative total spherical aberration (µm) | 0.10 ± 0.13 (-0.21 ~ 0.37) |
| Postoperative anterior chamber depth (mm) | 4.54 ± 0.51 (3.87 ~ 5.99) |
| Postoperative total spherical aberration (µm) | 0.02 ± 0.09 (-0.21 ~ 0.15) |
| Residual total spherical aberration (µm) | 0.08 ± 0.15 (-0.31 ~ 0.30) |
| IOL power (diopters) | 17.55 ± 5.52 (1.0 ~ 24.5) |

Table 1. Demographic data.
