# Supplementary material for: The efficiency of aspheric intraocular lens according to biometric measurements
Source: PLoS One. 2017 Oct 16;12(10):e0182606. doi: 10.1371/journal.pone.0182606 (PMC5642893; doi:10.1371/journal.pone.0182606)
Supplement: S2 Table — (DOCX) [file pone.0182606.s002.docx]

Table 2. Preoperative biometric data (axial length, anterior chamber depth, and central corneal power) and implanted IOL power in 2 subgroups stratified by internal spherical aberration.

|  | Internal SA  ≤ -0.06 µm | Internal SA  > -0.06 µm | *p* value$\dagger$ |
| --- | --- | --- | --- |
| No. of eyes | 23 | 17 |  |
| Preoperative axial length | 23.89 ± 1.31 | 25.40 ± 2.58 | 0.020 |
| Preoperative anterior chamber depth | 3.08 ± 0.41 | 3.17 ± 0.52 | 0.555 |
| Preoperative central corneal power | 44.84 ± 1.06 | 44.55 ± 1.52 | 0.487 |
| Preoperative total spherical aberration | 0.11 ± 0.06 | 0.10 ± 0.04 | 0.874 |
| Posoperative anterior chamber depth | 4.39 ± 0.33 | 4.74 ± 0.64 | 0.029 |
| Postoperative total spherical aberration | -0.02 ± 0.09 | 0.08 ± 0.05 | <0.001 |
| Residual total spherical aberration | 0.12 ± 0.13 | 0.02 ± 0.15 | 0.041 |
| Intraocular lens power | 18.93 ± 3.06 | 15.68 ± 7.46 | 0.040 |
| Age | 63.13 ± 10.04 | 62.47 ± 10.50 | 0.671 |
| Spherical aberration | -0.13 ± 0.05 | -0.03 ± 0.03 | <0.001 |

$\dagger$*p* value by Mann-Whitney U test
